# A borderline personality assessment for adolescents: Validity and reliability of the Chinese languages borderline personality features scale (short form version) for adolescents/children

**DOI:** 10.3389/fpsyt.2022.1050559

**Published:** 2022-12-15

**Authors:** Chuanjun Zhuo, Guangdong Chen, Chongguang Lin, Feng Jia, Lei Yang, Qiuyu Zhang, Jiayue Chen, Hongjun Tian, Deguo Jiang

**Affiliations:** ^1^Department of Psychiatry, Wenzhou Seventh Peoples Hospital, Wenzhou, China; ^2^Department of Psychiatry, Tianjin Fourth Center Hospital, Nankai University Affiliated Tianjin Fourth Center Hospital, Tianjin, China; ^3^PNGC_Lab, Tianjin Anding Hospital, Tianjin Mental Health Center of Tianjin Medical University, Tianjin, China

**Keywords:** Borderline Personality Features Scale for Children-Short Form, Chinese version, borderline personality disorder, validity, reliability

## Abstract

**Background:**

Borderline personality disorder (BPD) is characterized by behavioral patterns that promote suffering in many adolescents and their guardians. Currently, early diagnosis of BPD mainly depends on the effective assessment of pathological personality traits (i.e., borderline personality features) and using the indicated scales. The Borderline Personality Features Scale for Children-Short Form (BPFSC-SF) is widely used and the introduction of a Chinese version of the BPFSC-SF, can improve the diagnosis and prognosis of Chinese patients with BPD.

**Objective:**

The aim of the present study was to assess the validity and reliability of the Chinese version of the BPFSC-SF.

**Method:**

120 adolescents with BPD were enrolled in the present study and completed the BPFSC-SF and the Personality Belief Questionnaire-Short Form (PBQ-SF) assessments. Confirmatory factor analysis (CFA) was used to test assessment validity. Test-retest correlations and the Cronbach's α coefficients were used to determine reliability.

**Results:**

CFA analysis identified primary factors of BPFSC, with each item ranging from 0.597~0.899. The Spearman rank correlation coefficient was 0.877 between CL-BFSFC-SF and the state vs. trait loneliness scale. The Cronbach's α of the scale was 0.854 in the clinical group. The test-retest reliability correlation coefficient (interclass correlation coefficients.ICC) was 0.937.

**Conclusion:**

The Chinese version of BPFSC-SF is a valid and reliable tool for adolescent Chinese patients with BPD.

## Introduction

According to the DSM-IV criteria, borderline personality disorder (BPD) refers to behavioral personality patterns characterized by altered self-image and interpersonal relationships. BPD also encompasses a range of cognitive and emotional behavioral disturbances (1, 2). Although debated, some studies suggest that the diagnosis of personality disorders in childhood or adolescence, should be avoided and personality disorders must not be diagnosed in adolescence owing to continued personality maturation and high developmental variability (3, 4). More notably, many studies proposed that adolescence is a pivotal period in the development of personality disorder (5–12). For example, Cohen et al. reported that the onset of personality disorders emerges early in adolescence (13). The abovementioned results converged to indicated that some characteristics of adolescence might facilitate the onset of personality disorder (14, 15); specifically, these studies indicated that BPD may have predisposing features which facilitate the development of personality disorders (6, 10–15).

Some studies recommend that for adolescents with BPD, early diagnosis and intervention are ideal (3, 16–18) and provide optimal outcomes (19–22). More importantly, many studies have proposed that early diagnosis of BPD depends on careful and accurate assessment of pathological personality traits in adolescents (1, 23–26). Hence, ideal assessment scales, with good validity and reliability, can facilitate comprehensive clinical evaluations. However, to the best of our knowledge, there are no ideal assessments for BPD, specifically for Chinese adolescents. Previous studies have reported that the Borderline Personality Features Scale for Children-Short Form (BPFSC-SF) shows ideal validity and reliability when used to assess the BPD in the adolescents (27, 28), but its extended use has not been validated.

Effectiveness and feasibility of the assessments used for diagnosis plays a pivotal role in improving the long-term prognosis of patients with schizophrenia. Among the assessments used to assess schizophrenia, information regarding disease characteristics is gathered and widely used to inform a suitable plan aimed at improving the treatment outcome of patients with schizophrenia. However, rare assessment tools were used to assess the side effects of the patients with schizophrenia which induced by antipsychotic agents. Indeed, side effects may induce adverse events and may even be life threatening (29–32). The updated guidelines for clinical treatment of schizophrenia proposed that doctors address the side effects induced by antipsychotics since adverse treatment events can reduce treatment compliance (33). Hence, treatment side effects and treatment efficacy are related, due to this reason, ideal side effect assessment should be used in clinical practice.

BPFSC-SF, has widely been used as a preferred assessment tool for evaluating the side effects of antipsychotic agents and has greatly furthered our understanding. Accordingly, introduction of a Chinese version of the BPFSC-SF is an urgent task for Chinese psychiatrists. In the present study, we tested the validity and reliability of the Chinese version BPFSC-SF (CL-BPSFC) for use in Chinese adolescents with personality dysregulation. Our goal is to provide a useful tool for assessing the severity of personality problems, with the ultimate aim of improving the prognosis of adolescents with personality disorders.

## Materials and methods

### Participants

Patients were recruited from the departments of psychological consultation of Wenzhou Seventh Peoples' Hospital and of Tianjin Fourth Hospital, between May 2021 and May 2022; all patients met the DSM-IV diagnostic criteria for schizophrenia and were recruited by convenience sampling. A total of 120 patients were enrolled. The patients aged from 10 ~17 years old (average 12.31 ± 1.75 years). Personality disorder or duration of schizophrenia ranged from 6 ~ 19 months (average 10.25 ± 1.7 months). The ethics committee of Tianjin Fourth Center Hospital approved this study (No.2020-JW-117). All of the guardians included had participated in psychological counseling at least once in the past years. The inclusion criteria as follows: (1) IQ ≥ 80,2) can understanding the content of CL-BPSFC. 3) at least one time visit the crisis intervention center due to the personality problem in the last years. The exclusion criteria as follows: (1) with a history of family member had mental disorders, (2) with a history of patients had personality disorders, (3) with a history of brain disease, (4) with a history of severe physical disease.

### BPFSC-SF

BPFSC-SF comprises 11 items, including the following: (1) I feel very lonely; (2) I want to let some people know how much they have hurt me; (3) My feelings are very strong. For instance, when I get mad, I get really mad. When I get happy, I get really happy; (4) I am careless with things that are important to me; (5) People who were close to me have let me down; (6) I go back and forth between different feelings, like being mad or sad or happy; (7) I get into trouble because I do things without thinking; 8) I worry that people I care about will leave and not come back; (9) How I feel about myself changes a lot; (11) Lots of times, my friends and I are really mean to each other. Likert-type scale was used and defined as follows: None count 0; very little count 1; little count 2; frequently count 3; very frequently count 4.

### Localization and optimization

We translated the scale into Chinese and invited a native English speaker (S. Patricia Chou, chief of the NIAAA, https://www.niaaa.nih.gov/) to back-translate the Chinese languages-BPFSC-SF (CL-BPFSC-SF) to an English-language version. A final version of the CL-BPFSC-SF was acquired from the harmonized English-language version.

### Validity evaluation

Confirmatory factor analysis was used to determine structural validity; specifically, the variance maximum method was used to calculate the factors and their respective load. Spearman rank correlation coefficients were used to determine criterion validity; the state vs. trait loneliness scale (34) was adapted to the criterion.

### Reliability evaluation

A total of 120 participants were assessed independently by 9 raters. All raters knew the patient diagnoses but were blinded to each other's scores. The internal correlation coefficient (ICC) was used to assess the inter-consistency (35) and the Cronbach α coefficient of the full sample was used to determine split-half reliability (36).

### Defining of the cutoff point

Cutoff points were determined by taking the clinical standard provided by the consensus of 10 professional doctors working on treating personality disorders for over 20 years. The area under the receiver operating characteristic curve (AUC) acceptable (37) to the subjects, was judged as the cutoff point for the severity of borderline personality features, and then calculated the sensitivity and specificity of different CL- BPFSC-SF scores to evaluate the severity of borderline personality features.

### Statistical analysis

The relationship between BPFSC-SF score and state vs. trait loneliness scale score was analyzed by the Pearson correlation test. The internal consistency of the scale was evaluated by calculating the Cronbach's α coefficient and ICC. Confirmatory factor analysis was used to determine structural validity, the RMSEA was expected to be below 0.05, and the CFI, GFI and TLI were expected to be above 0.95 in order to be considered acceptable of the model fit by confirmatory factor analysis. Statistical significance was accepted as *p* < 0.05, within the 95% confidence interval (95% CIs) (35–37). SPSS software (IBM, version 20.0) was used for the statistical analyses of all variables.

## Results

### Construct validity

A confirmatory factor analysis revealed one principal factor among the 11 items which accounted for 87.00%, and was higher than the standard 50% of the structural validity test. Promax rotation demonstrated that each item had a high factor load (0.477–0.984; Table 1) (38).

**Table 1:** Item's factor loading of the CL- BPFSC-SF.

**Items**	**Factor loading, 95%CIs**	**Z**	***P***
1	0.671, 0.478–0.730	12.880	≤ 0.001
2	0.597, 0.444–0.824	14.589	≤ 0.001
3	0.617, 0.487–0.703	13.555	≤ 0.001
4	0.687, 0.444–0.824	16.274	≤ 0.001
5	0.766, 0.499–0.911	16.157	≤ 0.001
6	0.690, 0.452–0.767	14.599	≤ 0.001
7	0.874, 0.602–0.999	24.330	≤ 0.001
8	0.702, 0.511–0.901	18.997	≤ 0.001
9	0.785, 0.634–0.956	16.714	≤ 0.001
10	0.889, 0.699–0.999	20.369	≤ 0.001
11	0.755, 0.499–0.855	25.677	≤ 0.001

CL-BPFSC-SF, Chinese version of the Borderline Personality Features Scale for Children-Short Form; CI, confidence interval.

### Criterion validity

The Spearman rank correlation coefficient was 0.877 between CL-BFSFC-SF and the state vs. trait loneliness scale.

### Reliability data

Inter-rater consistency: The total ICC value of the inter-rater consistency was 0.854, indicating that the scale had good adaptability. The Cronbach α coefficient of the total scale was 0.937 (35–37).

### Validity data

A single factor structure was not determined in our study on the 11-item CL-BFSFC-SF. The sample data was suitable for factor analysis based on the Kaiser-Meyer-Olkin (KMO) measure and Bartlett's test of sphericity. In this study, a KMO of 0.87 and Bartlett's χ^2^ value of 2,898.74 (*P* < 0.01) met the conditions for confirmatory factor analysis (CFA), and the cumulative variance contribution rate was 72.530%. The CEFA was conducted using the maximum variance method to evaluate each item results, which showed that all the items were more than 0.400 (Table 1).

Taking the clinical evaluation standard of personality features as a reference, ROC demonstrated that the cutoff score was ≥42, accompanied with a sensitivity of 0.977 and specificity of 0.874 (AUC is 0.882). Compared to the clinical cutoff definition of 44 which suggests a severe personality feature, the cutoff score used was ≥30, accompanied with a sensitivity of 0.904 and specificity of 0.929 (AUC was 0.783). Clinically, 30 is used as the cutoff to define a moderate personality feature; the cutoff score used was ≥14, accompanied with a sensitivity of 0.980 and specificity of 0.888 (AUC is 0.857). Compared to the clinical definition, cutoff point is 14, the definition suggested a mild personality feature (Table 2).

**Table 2:** Cutoff scores of CL-BPFSC-SF.

**Cutoff score**	**Sensitivity**	**Specificity**	**AUC, 95% CIs**	***Z***	***P***
42	0.977	0.874	0.882, 0.711–0.932	18.361	≤ 0.001
30	0.804	0.929	0.783, 0.665–0.849	20.534	≤ 0.001
14	0.980	0.888	0.857, 0.700–0.994	23.870	≤ 0.001

CL-BPFSC-SF, Chinese version of the Borderline Personality Features Scale for Children-Short Form; AUC, area under curve; CI, confidence interval.

## Discussion

The results from this study confirmed that CL- BPFSC-SF has good validity and reliability and can be used as an assessment tool to evaluate adolescents with personality disturbances. Data provided by ROC analysis indicated that CL- BPFSC-SF can be used to evaluate the severity of personality problems in Chinese adolescents. Validity is a very important feature of any assessment and good validity can provide more precise information for clinical applications. The construct validity of the CL- BPFSC-SF was supported by confirmatory factor analysis. A KMO of 0.87 and Bartlett's χ^2^ value of 2898.74 (*P* < 0.01), and the cumulative variance contribution rate of 82.53%, indicated that the constructive validity of CL- BPFSC-SF is sufficient to be used as an assessment tool. The Spearman rank correlation coefficient was 0.877 between the CL-BFSFC-SF and the state vs. trait loneliness scale; these data demonstrated that CL-BFSFC-SF confers ideal criterion validity. Overall, the correlation with the state vs. trait loneliness scale suggests that the CL-BFSFC-SF can be used to assess the severity of aberrant personality features.

Effective reliability can provide more consistent information for screening individuals with specific characteristics. Our data demonstrated that the inter-rater consistency gained from the ICC and the split-half reliability gained from Cronbach α coefficient analysis all converged to support that CL-BFSFC-SF had efficient reliability. Notably, by using the ROC method, our data stratified the scores of CL-BFSFC-SF to discriminate the mild, moderate, severe personality features. Taken together and based on its reliability, the BFSFC-SF can be used as an ideal tool to assessment the personality features.

Over the last decade, many studies have employed the BFSFC-SF to screen personality features of adolescents (39–43). For example, Biberdzic et al. used the BFSFC-SF to assess adolescents' core domains of functioning; Barkauskiene et al. used BFSFC-SF to screen adolescent borderline personality features to provided information for the established DSM-V. Hendriks et al. used the BFSFC-SF to explore the psychopathological correlates of implicit and explicit shame and guilt; Sharp et al. used the BFSFC-SF to investigate maladaptive identity formation in adolescence. These studies suggested that the BFSFC-SF was a suitable tool to screen adolescent personality features. We now extend those findings and suggest that the CL-BSFC has high validity and reliability and can effectively be used in Chinese.

### Limitations

Our study design is not without limitation. We only enrolled adolescents who had been diagnosed with BPD by the hospital. Hence, the lack of a healthy control (due to the stigma of mental health, healthy “control” adolescents are very difficult to recruit) is a major flaw of this study. In future work, we will overcome difficulties in recruiting healthy controls to participate in the study and modify the CL-BSFC-SF to provide a good assessment tool for evaluating Chinese adolescents.

### Conclusions

Our data reveal that the Chinese Languages Borderline Personality Features Scale (Short Form Version) had ideal validity and reliability and it can be used for the assessment of Chinese adolescent borderline personality features. To the best of our knowledge, very few scales are available for the assessment of borderline personality features in Chinese adolescents. Our study would pave the way for new research on borderline personality features in adolescents and facilitate a more effective application of already available assessments.

## Data availability statement

The original contributions presented in the study are included in the article/supplementary material, further inquiries can be directed to the corresponding authors.

## Ethics statement

The studies involving human participants were reviewed and approved by the Ethics Committee of Tianjin Fourth Center Hospital. The patients/participants provided their written informed consent to participate in this study.

## Author contributions

All authors listed have made a substantial, direct, and intellectual contribution to the work and approved it for publication.

## Appendix

**The scale of CL-BPFSC-SF**

中文版青少年边缘人格评估量表（简版）

评分原则**:** 在过去的 **2** 年中, 您存在以下感觉吗？、

如果有, 记为 **0** 分, 如果有, 记为 **1**。

如果存在以下感觉, 则再次量化统计如下**:**

**1**分, 很少时间（一周内有**1**天时间左右）；

**2**分, 一部分时间（一周内有**2-3**天时间左右）；

**3**分, 大部分时间（一周内有**3-4**天时间左右）；

**4**分, 绝大部分时间（一周内有**5-6**天时间左右）；

**5**分, 一直存在（一周内 **7**天时间全部存在）。 条目如下**:**

**1**. 我感觉到非常孤独。

**2**. 我想让有些人知道, 他们对我的伤害有多深。

**3**. 我的情绪起伏很大。例如, 有时我会感觉到我疯了, 我真的疯了。有时我会感觉到我莫名其妙的亢奋, 而且亢奋的过头了。

**4**. 我感觉到我丢失了对我很重要的东西, 但是具体是什么东西, 我不知道。

**5**. 我不关心事情对我重要不重要（尽管有时候有些事情很重要）。

**6**. 对于我亲近的人, 我并不感到亲近。

**7**. 我在不同的情绪之间转变很快。例如, 我会突然从歇斯底里状态转化到悲伤状态, 或者转化到亢奋状态。

**8**. 有时候我会因为做事不过脑子而惹上很多麻烦。

**9**. 我担心我关心的人会抛弃我。

**10**. 我能感觉到我的情绪起伏很大。

**11**. 很多时候, 我和我的朋友对彼此都很刻薄。
